# Improving the resolution of canine genome-wide association studies using genotype imputation: A study of two breeds

**DOI:** 10.1111/age.13117

**Published:** 2021-07-12

**Authors:** Christopher A. Jenkins, Dog Biomedical Variant Database Consortium, Ellen C. Schofield, Cathryn S. Mellersh, Luisa De Risio, Sally L Ricketts

**Affiliations:** * Department of Veterinary Medicine, Kennel Club Genetics Centre^1^, University of Cambridge, Cambridge, UK.; † Division of Population Health, Health Services Research & Primary Care, University of Manchester, Manchester, UK.; ‡ Neurology/Neurosurgery Service, Centre for Small Animal Studies, Animal Health Trust, Newmarket, Suffolk, UK.

**Keywords:** Border Collie, genome-wide association study, imputation accuracy, Italian Spinone, whole genome sequencing

## Abstract

Genotype imputation using a reference panel that combines high-density array data and publicly available whole genome sequence consortium variant data is potentially a cost-effective method to increase the density of extant lower-density array datasets. In this study, three datasets (two Border Collie; one Italian Spinone) generated using a legacy array (Illumina CanineHD, 173 662 SNPs) were utilised to assess the feasibility and accuracy of this approach and to gather additional evidence for the efficacy of canine genotype imputation. The cosmopolitan reference panels used to impute genotypes comprised dogs of 158 breeds, mixed breed dogs, wolves and Chinese indigenous dogs, as well as breed-specific individuals genotyped using the Axiom Canine HD array. The two Border Collie reference panels comprised 808 individuals including 79 Border Collies and 426 326 or 426 332 SNPs; and the Italian Spinone reference panel comprised 807 individuals including 38 Italian Spinoni and 476 313 SNPs. A high accuracy for imputation was observed, with the lowest accuracy observed for one of the Border Collie datasets (mean *R*^2^ = 0.94) and the highest for the Italian Spinone dataset (mean *R*^2^ = 0.97). This study’s findings demonstrate that imputation of a legacy array study set using a reference panel comprising both breed-specific array data and multi-breed variant data derived from whole genomes is effective and accurate. The process of canine genotype imputation, using the valuable growing resource of publicly available canine genome variant datasets alongside breed-specific data, is described in detail to facilitate and encourage use of this technique in canine genetics.

## Introduction

Genotype imputation is a computational method that predicts missing genotypes in a dataset of genotyped individuals, using a reference panel of individuals genotyped at a higher density (Marchini *et al*. 2007; Howie *et al*. 2009). Imputation can enable meta-analyses of data generated using different arrays that include differing sets of SNP markers, and can increase the resolution of genome-wide association study (GWAS) datasets by increasing SNP density and allowing inclusion of SNPs not genotyped on that array (Browning 2008). Genotype imputation is a well-established tool in human genetics, facilitated by the availability of large datasets of human genetic variation, such as the HapMap (The International HapMap Consortium *et al*. 2007); 1000 Genomes Project (The 1000 Genomes Project Consortium *et al*. 2012); and Haplotype Reference Consortium (McCarthy *et al*. 2016), that can be used as reference panels for imputation of GWAS array data (Howie *et al*. 2011). The reference panels used can include a mixture of both population-specific panels and more divergent and cosmopolitan panels. An inclusive approach, using a reference panel with a composite of individuals closely related to the study population and individuals from other populations, can improve imputation accuracy (Howie *et al*. 2011).

Genotype imputation has also been established in other mammalian species, such as horse (Corbin *et al*. 2014; McCoy & McCue 2014; Schaefer *et al*. 2017; Chassier *et al*. 2018), cattle (Hozé *et al*.2013; Pausch*etal*. 2013; Korkuć *etal*.2019), pig (Gualdrón Duarte*etal*.2013;vandenBerg*etal*.2019)and sheep (Hayes *et al*. 2012; Bolormaa *et al*. 2019). The feasibility of using genotype imputation in the domestic dog has also been demonstrated; examples include imputation from a theoretical very low-density array up to the commonly used Illumina CanineHD BeadChip array (Friedrich *et al*. 2018), and imputation from an array up to whole genome level (resulting in 4.9–24 million variants; Friedenberg & Meurs 2016; Hayward *et al*. 2019). Furthermore, genotype imputation has been shown to facilitate the identification of potentially novelloci for complex traits in dogs and the refining of intervals for known associated loci (Hayward *et al*. 2019).

It has been demonstrated that to impute genotypes accurately in the dog, several reference panel individuals specific to the breed of the dogs in the study set are required in combination with individuals of multiple other breeds (Friedenberg & Meurs 2016). Genome sequence consortia could be invaluable resources for this approach, particularly for the generation of a multi-breed reference panel (Jagannathan *et al*. 2019; Ostrander *et al*. 2019). Such consortia have produced large variant datasets that are, or will become, publicly available. However, genome consortia datasets may include relatively few dogs of each breed, and many of the less common breeds may not be represented at all. Despite the decreasing cost of whole genome sequencing, generating a breed-specific component of a whole genome reference panel may be unfeasible for smaller studies. In recent years, however, a new higher density genotyping array for the canine genome has become available: the Axiom Canine HD array, which genotypes over 710 000 markers. Genotyping a set of breed-specific individuals using this array for use in a reference panel for imputation is comparatively cost-effective. Before the development of the Axiom Canine HD array, the 173 662-SNP Illumina CanineHD array had been used extensively for research since 2011 (Lequarré *et al*. 2011), meaning long running and ongoing studies often have extant datasets generated using this array. Applying genotype imputation to bring existing datasets up to marker densities comparable with the newer Axiom array could be an attractive way to utilise the wealth of data already available and increase the resolution and concomitant power of datasets.

There is still a need to build evidence for the optimum size of the breed-specific component of canine reference panels, and to examine how this may vary by breed. To date there is also a scarcity of literature outlining in detail the process of imputation in the dog, and to the authors’ knowledge no publications describing in detail the imputation of canine genotypes from the commonly used high density Illumina array up to the newer and increasingly utilised higher density Axiom array. This knowledge would be highly valuable to many researchers without the resources to generate large whole genome sequencing (WGS) datasets, with current WGS consortia containing only limited numbers of individuals of most breeds. This study intended to address these points and to provide further evidence towards a best practice method for accurate imputation in the dog.

The aim of the present study was to validate the use of genome-wide genotype imputation to impute extant Illumina CanineHD datasets up to the genotype density possible through the Axiom Canine HD array. Three Illumina datasets of two different breeds (two Border Collie and one Italian Spinone dataset) were imputed, assessing the effect of breed and reference panel size on imputation accuracy.

## Materials and methods

**Figure 1:**
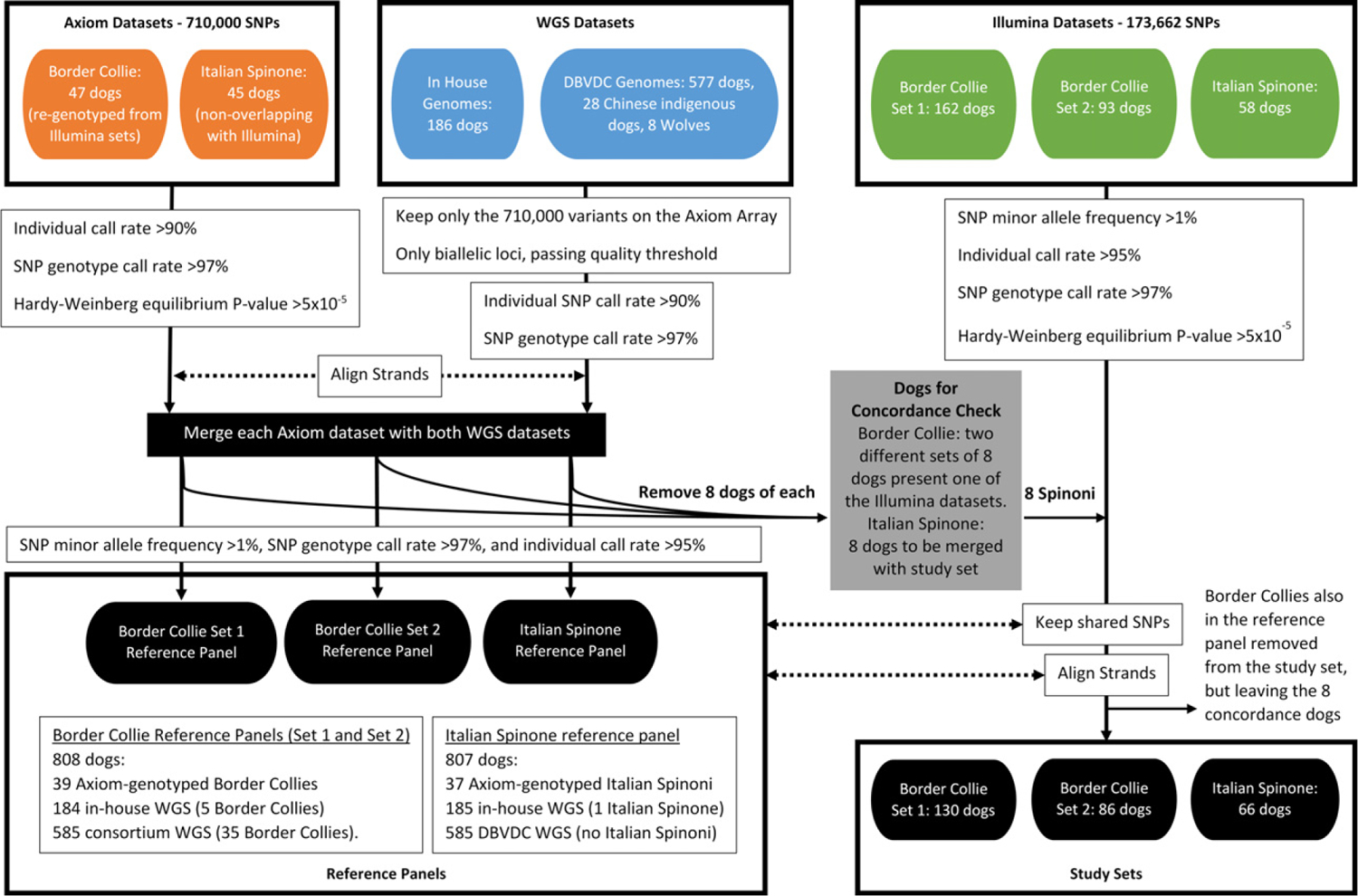
Flowchart to illustrate dataset processing for imputation study sets and reference panels. WGS, whole genome sequencing

The steps involved in preparing datasets for imputation, and the datasets used, which are described in detail below, are summarised in Fig. 1. For this study, each reference panel was assembled using data from three datasets: a breed-specific dataset (either Border Collie or Italian Spinone) genotyped using the Axiom Canine HD array; and two sets of array marker data extracted from WGS datasets (one in-house WGS dataset including 186 dogs of multiple breeds, and a consortium (Dog Biomedical Variant Database Consortium, DBVDC) WGS dataset comprising 577 dogs of multiple breeds, 28 Chinese indigenous dogs and eight wolves). These reference panels were used to impute Axiom genotypes in three study sets that had been genotyped using the Illumina CanineHD array (‘Border Collie Set 1’, ‘Border Collie Set 2’ and ‘Italian Spinone’; Fig. 1).

### Array-genotyped datasets for breed-specific reference panels

The Axiom Canine HD array genotype datasets, one each for the Italian Spinone and Border Collie breeds, were processed for quality control using the Axiom Analysis Suite and the Best Practices Workflow. Genotype data were available for 47 dogs (579 158 SNPs) in the Border Collie dataset, and 45 dogs (593 264 SNPs) in the Italian Spinone dataset (Fig. 1).

### WGS for the multi-breed reference panel

As stated above, two sets of WGS were used to make up the multi-breed component of the reference panel (Fig. 1). The first set consisted of 186 in-house WGS of dogs, representing 93 breeds and five mixed breed dogs, accrued over time for other research and as a resource (average coverage >30×, lowest coverage 11×; Table S1). The second set was an international consortium (DBVDC) dataset that included sequence variant data for an additional 577 dogs (117 breeds, in addition to mixed breed dogs), eight wolves and 28 Chinese indigenous dogs (Jagannathan *et al*. 2019). The genomes included in the DBVDC had an average of approximately 24× coverage, and a minimum of 10× coverage.

The Axiom Canine HD array SNPs were extracted from the two sets of WGS variant data using VCFTOOLS (v0.1.15; Danecek *et al*. 2011) to allow the data to eventually be merged with the breed-specific Axiom array genotype data. A minimum quality score (minQ) was set to 20 to exclude genotypes with quality scores (Phred) below this threshold, and only biallelic loci were extracted. The output files produced by VCFTOOLS were in PLINK ped and map format (Purcell *et al*. 2007).

### Aligning variant datasets from the Axiom array and WGS

Genotype data from both WGS datasets (in-house WGS dataset and DBVDC WGS dataset), and each Axiom dataset, were filtered using PLINK (v1.07) to exclude individuals genotyped for <90% of the SNPs, and to exclude SNPs that were called in <97% of individuals. Axiom datasets were also filtered to exclude SNPs with a Hardy–Weinberg equilibrium *P*-value <5 × 10^−5^ (Fig. 1). None of the datasets were filtered by minor allele frequency (MAF) at this stage to retain as many SNPs as possible prior to merging.

The statistical software package stata (Stata 15) was used to identify genotypes with strands that did not match between the datasets, and SNPs or variants which were insertions, deletions, or not biallelic across the datasets. Although only strand-flipped SNPs that were not between complementary bases (i.e. T/C, A/G) could be identified using this method, the small number that were found (Border Collie: *n* = 78; Italian Spinone: *n* = 91) indicated that the number of missed flipped SNPs is likely to be negligible. The identified insertions, deletions and SNPs that were not biallelic were excluded, and the strands of the strand-flipped SNPs were aligned using plink (v1.07).

### Merging the datasets to make a reference panel

For each of the two breeds, a combined reference panel was created using the Axiom array marker variants extracted from the two sets of WGS and the appropriate breed-specific Axiom canine HD array genotype dataset. To facilitate this, these three datasets were processed to keep only unique SNPs (i.e. removing SNPs within the same dataset that had different array IDs, but the same genomic position) that were present in all three (Fig. 1).

### Study sets

Two Border Collie GWAS sets (‘Border Collie Set 1’ included 162 dogs, ‘Border Collie Set 2’ comprised of 93 dogs) and one Italian Spinone set (58 dogs), all previously genotyped using the Illumina CanineHD array, were used in this research (Fig. 1). The Border Collie Illumina GWAS sets were genotyped at different times and therefore retained as separate study sets to preserve data quality and account for any between-run variability, as good practice for downstream use of the data in GWAS meta-analysis (Sung *et al*. 2014). Datasets were filtered to remove individuals with genotype call rates <95%, SNP call rates <97%, MAF <1% and Hardy–Weinberg equilibrium *P* < 5 × 10^−5^. A more stringent individual genotype call rate was used, in comparison to the initial filtering of the reference panel datasets, for consistency across chromosomes, to prevent individuals from later being removed by the filtering carried out for each chromosome prior to haplotype phasing. Only SNPs present in the corresponding reference panel were retained (Fig. 1).

### Dogs for analysing genotype concordance and imputation accuracy

The two Border Collie Illumina study sets contained dogs (33 in Set 1, 14 in Set 2) that were re-genotyped on the Axiom array (47 total) and which would therefore be part of the reference panel. All except eight of these re-genotyped dogs (selected at random to be kept in for use in calculating imputation accuracy and genotype concordance) were removed from each study set (Fig. 1). The two different sets of eight dogs for concordance calculations, one set for each Border Collie study set, were independently removed from the Border Collie reference panel. Each set of eight dogs was therefore present in one of the two original Illumina datasets, but there were no overlaps between each study set and its respective reference panel. This resulted in a different reference panel for each of the two Border Collie Illumina datasets (Fig. 1). For the Italian Spinone, there were no individuals present in both Illumina and Axiom datasets to use for assessing imputed genotype concordance. Instead, eight dogs genotyped using the Axiom array were selected at random to be excluded from the reference panel, filtered to leave only the SNPs present in the Illumina study set, and merged with this dataset (Fig. 1).

### Summary of the final reference panels

The pooled reference panels were filtered for SNP MAF <1%, SNP call rate <97% and individual call rate (<95%).

The final Border Collie reference panels were each comprised of 808 dogs: 39 Axiom-genotyped Border Collies; 184 in-house WGS (5 Border Collies); and 585 DBVDC WGS (35 Border Collies; Fig. 1). The Italian Spinone reference panel included 807 dogs: 37 Axiom-genotyped Italian Spinoni; 185 in-house WGS (1 Italian Spinone); and 585 DBVDC WGS (no Italian Spinoni). Each reference panel included dogs of 158 breeds, 12 mixed breed dogs, six wolves and 28 Chinese indigenous dogs.

To investigate the relationship between the number of breed-specific reference individuals and accuracy, two additional reference panels were produced for Border Collie Set 1, one without the 35 DBVDC Border Collies (‘44 Border Collie Reference Panel’) and a second with half of the in-house WGS and genotyped Border Collies removed at random (‘22 Border Collie Reference Panel’). The dogs were removed from the reference panel before filtering SNPs again as above.

### Multidimensional scaling plot of Set 1 Border Collies

To assess for the presence of any population stratification between the Axiom genotyped, in-house WGS and DBVDC Border Collies; and the Illumina genotyped Border Collies; a multidimensional scaling (MDS) plot of Border Collies included in the Border Collie Set 1 reference panel and study set was generated using PLINK (v1.90). The data for only the Border Collies was extracted from the Border Collie Set 1 reference panel and filtered to keep only the 100 535 SNPs also present in the Border Collie Set 1 study set. The resulting dataset was merged with the study set. The MDS plot included 39 Axiom-genotyped Border Collies, five in-house WGS Border Collies, 35 DBVDC WGS Border Collies and 130 Border Collie Set 1 study set dogs.

### Aligning study set variant datasets with reference panel variant datasets

The strands of the Illumina study set genotype data needed to be aligned with that of the reference panel before imputation could be carried out (Fig. 1). A considerable number of discrepancies were identified when comparing the Illumina strand annotations to those of the Axiom/WGS data. This could have been due to the Illumina CanineHD BeadChip probes being originally designed using the previous canine reference genome build BROADD2 whereas the Axiom Canine HD array and WGS were CanFam3.1. To identify all of the SNPs that needed to be strand flipped, flanking DNA information provided in the annotation documents for each of the two genotyping arrays was used. Ten bases of the upstream and downstream sequence for each of the SNPs were extracted from the annotation file and were compared between arrays. The strands of the study set SNPs that were not on the same strand between datasets were aligned (Fig. 1).

### Haplotype phasing and imputation

The reference panel and study sets were split by chromosome for haplotype phasing and imputation. Only the autosomes were used for imputation. Each individual in the reference panel and study set needed to pass a genotype rate threshold of 90% for each chromosome. Three individuals (originally part of the DBVDC WGS set) were excluded for the chromosome 9 (CFA 9) reference panel because they failed to pass this threshold.

The Border Collie Set 1 reference panel included 426 326 SNPs; and the Border Collie Set 2 reference panel included 426 332 SNPs. The Italian Spinone reference panel contained 476 313 SNPs. In the reduced Border Collie Set 1 reference panels, the number of SNPs was: 44 Border Collie Reference Panel, 426 235 SNPs; 22 Border Collie Reference Panel, 426 154 SNPs.

Haplotype phasing of reference panels and study sets was carried out using shapeit (v2, r904; Delaneau *et al*. 2012). Genotype imputation was carried out using impute2 (impute v2.3.2; Howie *et al*. 2009; Howie *et al*. 2011). A publicly available canine genetic map was used for haplotype phasing and imputation (Wong *et al*. 2010). A window size of 2 Mb was used for haplotype phasing, and the effective population size was set at 200 for both phasing and imputation (Friedenberg & Meurs 2016).

### Analysis of imputed genotypes

To assess accuracy of imputed genotypes, the predicted allele ‘dosage’ produced by imputation was compared to the ‘known’ genotypes in the array data for eight different dogs from each study set. After exclusion of the observed Illumina array genotypes, the squared Pearson correlation coefficient (*R*^2^) was calculated for each individual to give an indication of accuracy for each chromosome. Genotype concordance (%) was also calculated after converting the allele dosages provided by impute2 to binary genotypes using plink (v1.90; calls with uncertainty >0.1 were called as missing).

impute2 produces a metric, called Info, for each SNP that describes the reliability of the imputed genotypes. An Info score is a value typically between 0 and 1, with scores closer to 1 indicating greater certainty. The Info scores were split into 10 groups to allow visualisation of the data and comparison with previous studies, and the concordance of the SNPs with known heterozygous or homozygous genotypes in the eight dogs were analysed.

## Results and discussion

### Imputation accuracy and concordance, and comparison with previous studies

After filtering the SNPs as would typically be carried out for a GWAS (Hardy–Weinberg *P* < 5 × 10^−5^, call rate <97%, MAF <5%) the number available for analysis was on average (mean) three times higher than that of the study set (Table 1). This increase in SNP number and therefore density would be expected to reduce the gaps between genotyped SNPs, increasing the likelihood of a SNP tagging a risk-conferring variant in a GWAS (dependent on local LD structure). This also allows meta-analysis with data genotyped on the higher density Axiom array, without sacrificing a large proportion of the available data. However, the number of imputed SNPs is limited by the number within the reference panel, which is dependent on the allele frequencies within the breed. This can be seen clearly when comparing the relative sizes of the Border Collie and Italian Spinone reference panels and the number of SNPs in the resulting imputed datasets (Table 1).

Across the three imputed datasets, genotype dosages produced were highly correlated (>0.94) with the known genotypes provided by the array (Table 2). After conversion of the predicted dosages to binary genotype format, the percentage of genotypes concordant between the imputed data and array data was high (≥96.9%), demonstrating that genotype imputation was very accurate for all three datasets (Table 2). The concordances observed for the three sets imputed in this study are higher than that observed in a previous study also using impute2 but a smaller multi-breed reference panel to impute genotypes in Standard Poodles up to whole genome level (94.1%), and comparable to the same study’s results for the Boxer when using different software for imputation (Beagle 4.0, Browning & Browning 2007; 97.8%; Friedenberg & Meurs 2016). This previous study used a reference panel with a multi-breed component of 63 dogs representing 14 different breeds, and 19 breed-specific dogs (Standard Poodles or Boxers depending on the study set). When the breed-specific dogs were excluded from the study’s reference panel, or only dogs of other breeds were included, accuracy dropped. The present study utilised reference panels of over 800 dogs from 158 breeds (including breed-specific dogs), and accuracy was high for both Border Collies and Italian Spinoni. The inclusion of individuals in reference panels from other populations not matched to the study set (in addition to population-matched individuals) has also been shown to be effective for achieving optimum accuracy in the imputation of genotypes in human studies, by improving imputation of alleles less common in the study population, which may be poorly represented in population-matched individuals (Howie *et al*. 2011).

The concordance for the three sets in the present study was also higher than the highest concordance observed (92.7%) in another study that imputed genotypes of multiple dog breeds up to whole genome level using a multibreed reference panel of 365 WGS that included minimal (between 10 and 16) breed-specific dogs (Hayward *et al*. 2019). This highlights again the importance of breed-specific individuals in reference panels for canine genotype imputation accuracy. Including population-matched individuals has been demonstrated to be important for the accuracy of imputation of genotypes in human studies. Similarly, increasing the number of breed-matched individuals in reference panels can improve imputation accuracy in cattle (Hozé *et al*. 2013).

Both of the two aforementioned canine studies (Friedenberg & Meurs 2016; Hayward *et al*. 2019) imputed from the Illumina CanineHD array or a comparable array up to whole genome level, whereas the present study imputed up to the Axiom array, a comparatively lower proportion of SNPs. It is possible that imputing a greater proportion of SNPs increases error rate. However, previous work has suggested that it is the density of the known SNPs (the number of existing genotypes) in the study set that has the greatest impact on accuracy, not the number of missing SNPs that need to be imputed to bring the study set up to the size of the reference panel (Friedrich *et al*. 2018; Qanbari 2020). It could be that studies imputing to whole genome level impute a greater proportion of SNPs with low MAF. Alleles with the lowest frequencies are well established as having a reduced accuracy when imputed, particularly for heterozygous loci (Howie *et al*. 2011; Friedrich *et al*. 2018; Hayward *et al*. 2019).

### Variation in imputation accuracy across chromosomes and study individuals

**Figure 2:**
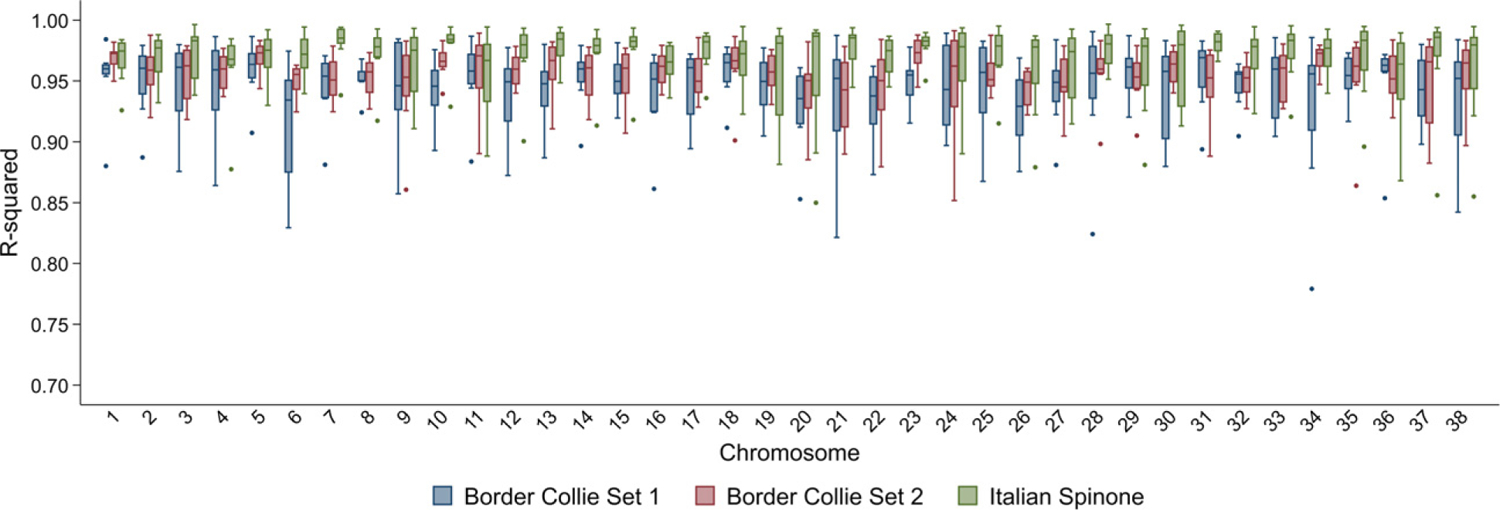
Accuracy of imputation for each chromosome in Italian Spinone and Border Collie datasets. The graph shows the *R*^*2*^ of imputed calls and known genotypes. Boxes are 25th–75th percentiles, with lines for the median. Whiskers indicate upper and lower adjacent values; outliers are shown using dots. Truncated *y*-axis starts at 0.7.

**Figure 3:**
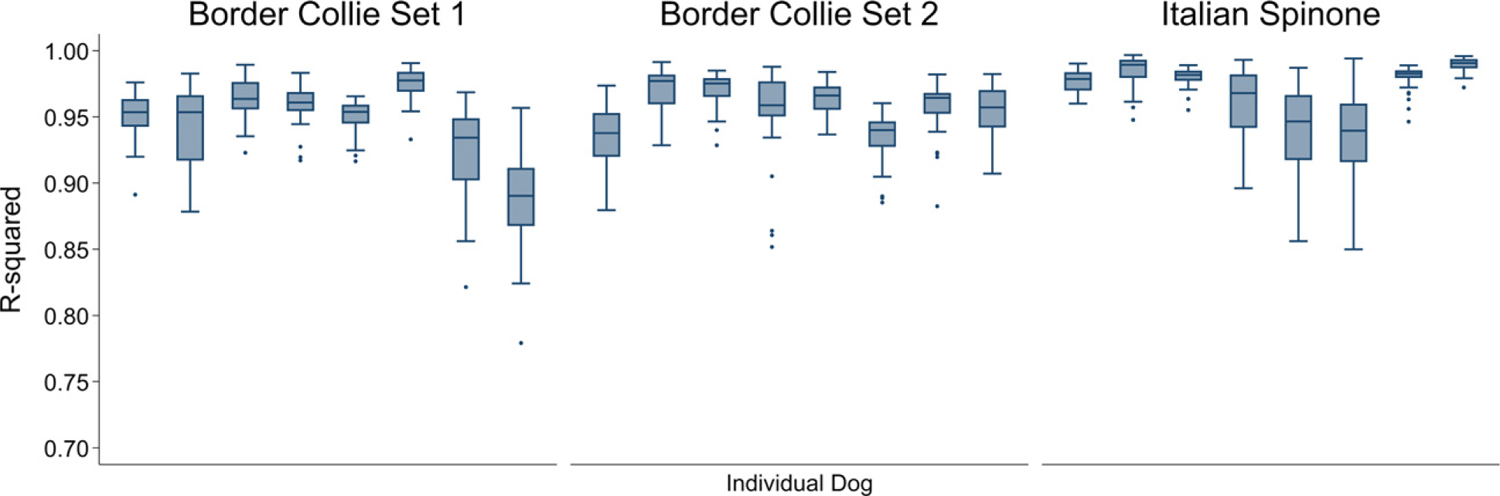
Accuracy of imputation for each concordance-tested individual (*n* = 8 for each set) in Italian Spinone and Border Collie datasets. The graph shows the *R*^*2*^ of imputed calls and known genotypes. Boxes are 25th–75th percentiles, with lines for the median. Whiskers indicate upper and lower adjacent values; outliers are shown using dots. Truncated *y*-axis starts at 0.7.

Accuracy was moderately consistent across autosomes, although some variation was observed (Table 2, Fig. 2). There was no correlation between chromosome size and imputation accuracy in this or a previous study (Friedenberg & Meurs 2016). However, a correlation between accuracy and chromosome size was seen in the other study that imputed up to genome level (Hayward *et al*. 2019). Imputation accuracy was also variable across individuals (Table 2, Fig. 3). Border Collie Set 1 showed the biggest difference in mean *R*^2^ values between the individuals (and, to a lesser extent, chromosomes) with the highest and lowest accuracies.

### Study-specific differences and the effect of reducing the number of breed-specific reference panel individuals on imputation accuracy

**Figure 4:**
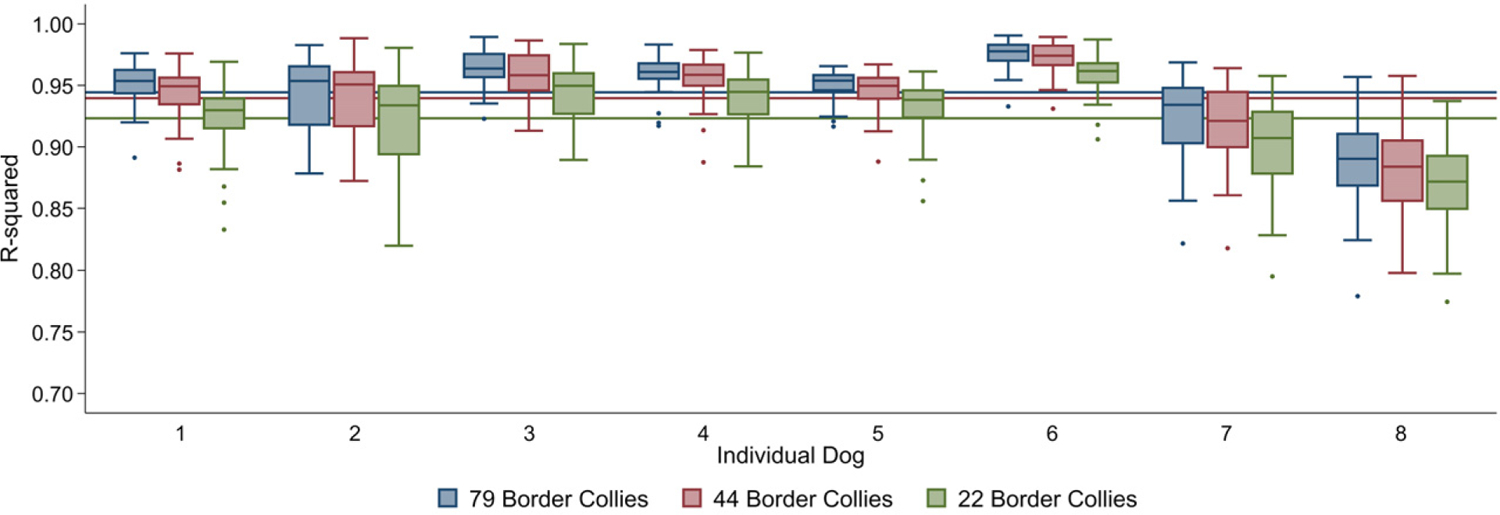
Accuracy of imputation for each concordance tested dog from Border Collie Set 1 and each of three reference panels containing decreasing numbers of Border Collies. The graph shows the *R*^*2*^ of imputed calls and known genotypes. Boxes are 25th–75th percentiles, with lines for the median. Whiskers indicate upper and lower adjacent values; outliers are shown using dots. Lines show mean *R*^*2*^ for each reference panel. Truncated *y*-axis starts at 0.7.

Border Collie Set 1 had the lowest imputation accuracy, and the highest accuracy was observed for the Italian Spinone dataset (Table 2, Fig. 2), despite the Italian Spinone reference panel including only 38 breed-specific dogs, whereas the Border Collie reference panels contained more than double the number (79 Border Collies). This indicates that the relationship between accuracy and the size of the breed-specific component of the reference panel reaches a plateau, and that other factors also have a role. To test this hypothesis, Border Collie Set 1 was imputed using two other reference panels: one without any of the DBVDC Border Collies (‘44 Border Collie Reference Panel’), and one with half of the remaining Border Collies (‘22 Border Collie Reference Panel’; Fig. 4). The 44 Border Collie Reference Panel did not materially reduce imputation accuracy (*R*^2^ = 0.94; Fig. 4). Using the 22 Border Collie Reference Panel had a greater effect, bringing the accuracy down to *R*^2^ = 0.92 (Fig. 4). This suggests that above 44 breed-specific dogs in the reference panel, imputation accuracy plateaued for the Border Collie, and that other factors caused this dataset to be imputed at a lower accuracy than the Italian Spinone set. The multi-breed reference panel used in this study included more dogs from more breeds than those described for previous studies (Friedenberg & Meurs 2016; Hayward *et al*. 2019); therefore, it is possible that the large number and diversity of haplotypes present limited the effect of reducing the number of breed-specific dogs on accuracy. Since differences between the levels of inbreeding and LD in the Border Collie and Italian Spinone breeds could also be contributing to some of the variation in accuracy observed; future work could compare imputation accuracy across many different breeds when using the same sized reference panel. The reduced accuracy in Border Collie Set 1 when compared to Border Collie Set 2 suggests differences in the sample populations or potentially lower DNA quality and therefore reduced genotype reliability in Set 1.

A study of imputation in sheep showed that including more closely related individuals in the reference panel can improve imputation accuracy (Hayes *et al*. 2012) and previous research has indicated that including related individuals can also increase accuracy in the dog (Friedrich *et al*. 2018), although the effect seen was minimal. The Border Collie breed is numerically much larger than the Italian Spinone, and the dogs included in the reference panel are therefore likely to be less closely related to those in the study set. The DBVDC is an international consortium, and the consortium Border Collies could therefore be expected to originate from populations less closely related to the study set, which were predominantly UK dogs, compared to the dogs used for array genotyping or WGS in the UK, which were also predominantly UK dogs. This could also partially explain why removing these dogs had only minimal effect on accuracy. To examine this, a MDS plot of Set 1 Border Collies (reference panel and study set) was generated using SNP data common to all four datasets (Axiom-generated Border Collies; in-house and DBVDC WGS-derived Border Collies; Illumina-genotyped Border Collie Set 1; Fig. S1). This demonstrated that the reference panel captures the study-set individuals effectively, and in particular that the combination of the Axiom and WGS-derived reference panels appears to give the greatest coverage of individuals. However, as the majority of the DBVDC individuals cluster with a close group (Fig. S1), it may be that the limited number of haplotypes in this group means that removing the DBVDC Border Collies had a smaller effect than removing a further 22 dogs, which may have been more distributed. Future research that examines imputation accuracy in breeds with known differences between geographical populations, such as the Retriever breeds (Arendt *et al*. 2015; Biasoli *et al*. 2019), would help to elucidate this.

Differences in the approaches used to calculate accuracy between the two breeds could also explain some of the differences observed. The dogs used to calculate concordance in the Italian Spinone dataset had been genotyped on the Axiom array before being filtered to keep only Illumina array SNPs before imputation. This created an artificial low-density dataset. By contrast, the Border Collies used to calculate concordance had been genotyped on both arrays, and the Illumina dataset imputed. Differences between accuracy of arrays, and errors in genotype calls when retesting, introduced discrepancies between the Border Collie datasets, whereas the Italian Spinone concordance dogs had identical genotypes between the reference panel and artificially created Illumina study set dogs. This means that accuracies are not directly comparable, although it does give an indication of the real differences.

### Imputation accuracy stratified by impute2’s imputation certainty (‘Info’) metric

**Figure 5:**
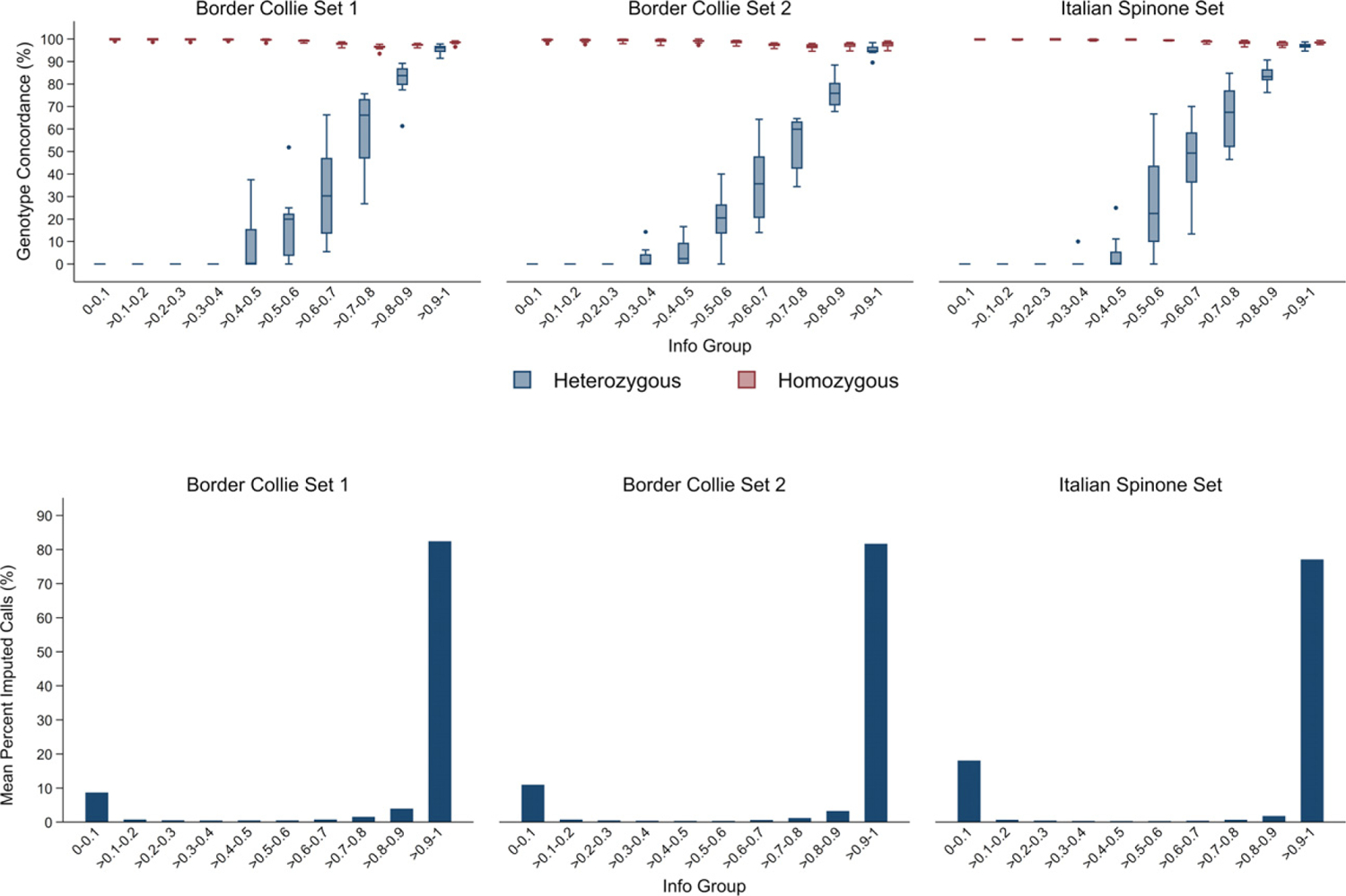
A comparison of imputation accuracy and predicted certainty. Top: percent of concordant genotypes for SNPs with heterozygous or homozygous known genotypes grouped by impute2’s Info metric (imputation certainty). Bottom: percent of total imputed calls within each Info group.

The accuracy of imputation across the range of the ‘Info’ statistic, split into 10 ‘Info groups’, was assessed. The concordance of homozygous SNPs was consistently high across the Info groups, but heterozygous genotypes had a low concordance in the lower Info groups (Fig. 5), consistent with earlier canine research (Friedenberg & Meurs 2016). Most SNPs fell within either the very lowest Info group or the higher Info groups, which is also similar to previously published findings (Friedenberg & Meurs 2016). When the grouped Info scores were compared to the expected allele frequency provided by the impute2 software, a positive trend was observed (Table S2); however, this was skewed by the lowest and highest Info score groups containing the majority of the SNPs with low frequency alleles (Table S3). The Info metric produced by impute2 can be used to filter the imputed SNPs to remove those for which there is a lower imputation certainty. The results from this study indicate that the optimum threshold to use for filtering by Info will vary depending on the breed of dog in the dataset imputed. A higher threshold might be necessary for the Border Collie, compared to the Italian Spinone, to ensure highest accuracy without excluding too many useful SNPs (Fig. 5). However, the majority of the SNPs with lower imputation certainty will be filtered out of downstream GWAS analyses by MAF (Tables S2 & S3).

## Conclusions

This research has demonstrated and described in detail the successful use of imputation to bring the SNP density of the commonly used Illumina array closer to that of datasets generated using the newer higher-density, and increasingly used, Axiom array. This represents a cost-effective method to make the most use of extant data, without the need to re-genotype all individuals or generate large WGS datasets as would be necessary for imputation up to the density of WGS, which has been the predominant focus of previous literature in the canine field. The present study demonstrates that in-house and publicly available consortium WGS variant datasets can be used to produce multi-breed reference panels large and diverse enough to enable accurate genotype imputation of canine GWAS datasets. This work contributes to building best practice evidence for the optimum size of the breed-specific component of canine reference panels, demonstrating that increasing the number of breed-specific dogs improves accuracy, and providing some initial evidence for the upper threshold, after which adding more dogs may have a limited effect. Although the number of breed-specific dogs required may vary significantly between breeds, our analysis of the Border Collie has shown that effective imputation can be carried out in a genetically diverse and numerically large breed using a modest number of breed- specific dogs in the reference panel. As well as investigating imputation in additional breeds, including those with distinct geographically isolated populations, it will be important for future applications to examine regions of gene complexity, such as the major histocompatibility complex, where imputation accuracy may be highly variable across breeds.

## Supplementary Material

Supplementary Material Table S1 The number of individuals for each of the 93 breeds, and mixed breeds, included in the dataset of 186 in-house WGS.
Table S2 Comparison of the expected frequency of the allele coded as ‘1’ (provided by impute2) for imputed SNPs across grouped Info scores for the three datasets.
Table S3 Comparison of the number of imputed SNPs with an expected frequency of the allele coded as ‘1’ (provided by impute2) lower than 0.05 across grouped Info scores for the three datasets.
Figure S1 Multidimensional scaling (MDS) plot of 39 Axiom genotyped Border Collies, five in-house WGS Border Collies, 35 DBVDC WGS Border Collies, and 130 Border Collie Set 1 individuals genotyped using the Illumina array.
Appendix S1 Affiliations and funding information for DBVDC members.

## Figures and Tables

**Table 1:** SNPs in each dataset before and after imputation.

Dataset	Study set dogs (*n*)	Study set SNPs (*n*)	Total SNPs after imputation (*n*)	SNPs passing quality control^1^ (*n*)
Border Collie Set 1	130	100 535	426 326	310 617
Border Collie Set 2	86	105 443	426 332	310 300
Italian Spinone set	66	104 432	476 313	341 854

1 Hardy–Weinberg P > 5 × 10^−5^, call rate > 97%, minor allele frequency > 5%.

**Table 2:** Imputation accuracy across the three study datasets.

Dataset	Mean *R*^2^	Genotype concordance (%)	Worst Chr (mean *R*^2^)	Best Chr (mean *R*^2^)	Individual lowest R^2 1^	Individual highest R^2 1^
Border Collie Set 1	0.94	96.9	CFA 6 (0.92)	CFA 5 (0.96)	0.89	0.98
Border Collie Set 2	0.96	97.7	CFA 21 (0.94)	CFA 23 (0.97)	0.93	0.97
Italian Spinone set	0.97	98.2	CFA 36 (0.95)	CFA 7 (0.98)	0.94	0.99

1 Lowest or highest *R*^*2*^ observed in an individual dog.

## Data Availability

The Italian Spinone and Border Collie Axiom Canine HD array genotype datasets, and the Axiom Canine HD array SNPs extracted from the in-house WGS data, are available for download (https://doi.org/10.5061/dryad.prr4xgxkj). The DBVDC variant data are available at the European Variation Archive, project ID PRJEB32865 (https://www.ebi.ac.uk/eva/?eva-study=PRJEB32865).
