# Supplementary material for: Improving the resolution of canine genome-wide association studies using genotype imputation: A study of two breeds
Source: Anim Genet. Author manuscript; Available in PMC 2021 Oct 13. (PMC8514152; doi:10.1111/age.13117)
Supplement: Supplementary Material — Table S1 The number of individuals for each of the 93 breeds, and mixed breeds, included in the dataset of 186 in-house WGS. Table S2 Comparison of the expected frequency of the allele coded as ‘1’ (provided by impute2) for imputed SNPs across grouped Info scores for the three datasets. Table S3 Comparison of the number of imputed SNPs with an expected frequency of the allele coded as ‘1’ (provided by impute2) lower than 0.05 across grouped Info scores for the three datasets. Figure S1 Multidimensional scaling (MDS) plot of 39 Axiom genotyped Border Collies, five in-house WGS Border Collies, 35 DBVDC WGS Border Collies, and 130 Border Collie Set 1 individuals genotyped using the Illumina array. Appendix S1 Affiliations and funding information for DBVDC members. [file NIHMS1745165-supplement-Supplementary_Material.docx]

Table S1. The number of individuals for each of the 93 breeds, and mixed breeds, included in the dataset of 186 in-house WGS.

| **Breed** | **Count** |
| --- | --- |
| Affenpinscher | 1 |
| Airedale Terrier | 2 |
| Alaskan Malamute | 1 |
| American Cocker Spaniel | 1 |
| Australian Shepherd | 1 |
| Basset Hound | 4 |
| Beagle | 3 |
| Bearded collie | 2 |
| Bedlington terrier | 1 |
| Berger Picard (Picardy sheepdog) | 1 |
| Bloodhound | 1 |
| Border Collie | 7 |
| Border Terrier | 9 |
| Boxer | 1 |
| Briard | 2 |
| Bull Terrier | 1 |
| Bulldog | 3 |
| Cairn Terrier | 1 |
| Cavalier King Charles Spaniel | 3 |
| Cesky Terrier | 1 |
| Chesapeake Bay Retriever | 1 |
| Chihuahua | 1 |
| Chinese Crested | 2 |
| Chow Chow | 1 |
| Corgi | 1 |
| Mixed Breed | 5 |
| Dalmatian | 1 |
| Dandie Dinmont | 4 |
| Dobermann | 2 |
| English Setter | 1 |
| English Springer Spaniel | 2 |
| Field Spaniel | 1 |
| Finnish Lapphund | 1 |
| Flat Coated Retriever | 4 |
| French Bull Dog | 4 |
| German Shepherd Dog | 1 |
| Giant Schnauzer | 5 |
| Glen of Imaal Terrier | 1 |
| Golden Retriever | 2 |
| Gordon Setter | 1 |
| Grand Basset Griffon Vendeen | 1 |
| Great Dane | 1 |
| Greyhound | 2 |
| Griffon Bruxellois | 1 |
| Irish Red and White Setter | 4 |
| Irish Setter | 2 |
| Irish Terrier | 1 |
| Irish Water Spaniel | 1 |
| Irish Wolfhound | 6 |
| Italian Spinone | 2 |
| Japanese Akita | 1 |
| Keeshond | 3 |
| Labrador Retriever | 3 |
| Lagotto Romagnolo | 1 |
| Lakeland Terrier | 1 |
| Lancashire Heeler | 2 |
| Large Munsterlander | 1 |
| Leonberger | 2 |
| Lhasa Apso | 1 |
| Maltese | 1 |
| Miniature Long-haired Dachshund | 2 |
| Miniature Poodle | 1 |
| Miniature Schnauzer | 2 |
| Miniature Wire Haired Dachshund | 1 |
| Newfoundland | 1 |
| Northern Inuit | 2 |
| Norwegian Buhund | 3 |
| Norwich terrier | 1 |
| Nova Scotia Duck Tolling Retriever | 1 |
| Old English Sheepdog | 1 |
| Otterhound | 2 |
| Papillon | 1 |
| Petit Basset Griffon Vendeen | 2 |
| Pug | 3 |
| Rottweiler | 1 |
| Rough Collie | 1 |
| Scottish Terrier | 5 |
| Shar Pei | 2 |
| Shetland Sheepdog | 1 |
| Shih Tzu | 1 |
| Siberian Husky | 3 |
| Skye Terrier | 2 |
| Smooth Collie | 1 |
| Soft-coated Wheaten Terrier | 2 |
| Staffordshire Bull Terrier | 1 |
| Swedish Vallhund | 1 |
| Tibetan Spaniel | 1 |
| Tibetan Terrier | 2 |
| Vizsla (smooth coat) | 4 |
| Vizsla (Wire-haired) | 1 |
| Weimaraner | 1 |
| Welsh Springer Spaniel | 7 |
| West Highland White Terrier | 1 |
| Whippet | 1 |

Table S2. Comparison of the expected frequency of the allele coded as ‘1‘ (provided by IMPUTE2) for imputed SNPs across grouped Info scores for the three datasets.

|  | **Border Collie Set 1** | | | **Border Collie Set 2** | | | **Italian Spinone** | | |
| --- | --- | --- | --- | --- | --- | --- | --- | --- | --- |
| **Info group** | Mean expected allele frequency | Standard deviation | SNPs (n) | Mean expected allele frequency | Standard deviation | SNPs (n) | Mean expected allele frequency | Standard deviation | SNPs (n) |
| 0-0.1 | 0.0006 | 0.02 | 27,743 | 0.0015 | 0.04 | 34,608 | 0.0031 | 0.06 | 66,315 |
| >0.1-0.2 | 0.0031 | 0.04 | 2,424 | 0.0070 | 0.07 | 2,337 | 0.0050 | 0.05 | 2,522 |
| >0.2-0.3 | 0.0053 | 0.05 | 1,665 | 0.0056 | 0.05 | 1,548 | 0.0087 | 0.07 | 1,644 |
| >0.3-0.4 | 0.0065 | 0.05 | 1,480 | 0.0105 | 0.08 | 1,251 | 0.0074 | 0.04 | 1,267 |
| >0.4-0.5 | 0.0131 | 0.08 | 1,524 | 0.0154 | 0.09 | 1,172 | 0.0149 | 0.08 | 1,173 |
| >0.5-0.6 | 0.0275 | 0.12 | 1,575 | 0.0290 | 0.12 | 1,173 | 0.0237 | 0.10 | 1,167 |
| >0.6-0.7 | 0.0466 | 0.15 | 2,524 | 0.0534 | 0.17 | 2,023 | 0.0317 | 0.11 | 1,533 |
| >0.7-0.8 | 0.0598 | 0.17 | 5,225 | 0.0744 | 0.19 | 3,960 | 0.0571 | 0.16 | 2,603 |
| >0.8-0.9 | 0.0877 | 0.20 | 13,238 | 0.1078 | 0.23 | 10,855 | 0.1098 | 0.22 | 6,882 |
| >0.9-1 | 0.3228 | 0.28 | 268,393 | 0.3257 | 0.27 | 261,962 | 0.3539 | 0.28 | 286,775 |

Table S3. Comparison of the number of imputed SNPs with an expected frequency of the allele coded as ‘1‘ (provided by IMPUTE2) lower than 0.05 across grouped Info scores for the three datasets. The percentage of the total number of imputed SNPs with an expected allele frequency lower than 0.05 that are within each Info group is shown.

|  | **Border Collie Set 1** | | **Border Collie Set 2** | | **Italian Spinone** | |
| --- | --- | --- | --- | --- | --- | --- |
| **Info group** | SNPs with expected allele frequency <0.05 (n) | Percent of all SNPs with expected allele frequency <0.05 (%) | SNPs with expected allele frequency <0.05 (n) | Percent of all SNPs with expected allele frequency <0.05 (%) | SNPs with expected allele frequency <0.05 (n) | Percent of all SNPs with expected allele frequency <0.05 (%) |
| 0-0.1 | 27,727 | 27.16 | 34,558 | 34.45 | 66,111 | 53.54 |
| >0.1-0.2 | 2,420 | 2.37 | 2,325 | 2.32 | 2,515 | 2.04 |
| >0.2-0.3 | 1,660 | 1.63 | 1,544 | 1.54 | 1,635 | 1.32 |
| >0.3-0.4 | 1,474 | 1.44 | 1,241 | 1.24 | 1,262 | 1.02 |
| >0.4-0.5 | 1,502 | 1.47 | 1,151 | 1.15 | 1,149 | 0.93 |
| >0.5-0.6 | 1,512 | 1.48 | 1,123 | 1.12 | 1,121 | 0.91 |
| >0.6-0.7 | 2,314 | 2.27 | 1,841 | 1.84 | 1,412 | 1.14 |
| >0.7-0.8 | 4,603 | 4.51 | 3,342 | 3.33 | 2,168 | 1.76 |
| >0.8-0.9 | 10,008 | 9.8 | 7,539 | 7.52 | 4,622 | 3.74 |
| >0.9-1 | 48,855 | 47.86 | 45,648 | 45.51 | 41,496 | 33.6 |

Figure S1. Multidimensional scaling (MDS) plot of 39 Axiom genotyped Border Collies, five in-house WGS Border Collies, 35 DBVDC WGS Border Collies, and 130 Border Collie Set 1 individuals genotyped using the Illumina array. MDS data are based on 100,535 SNPs and were generated using PLINK (v1.90). Triangles indicate individuals that were included in the ’22 Border Collie Reference Panel’.
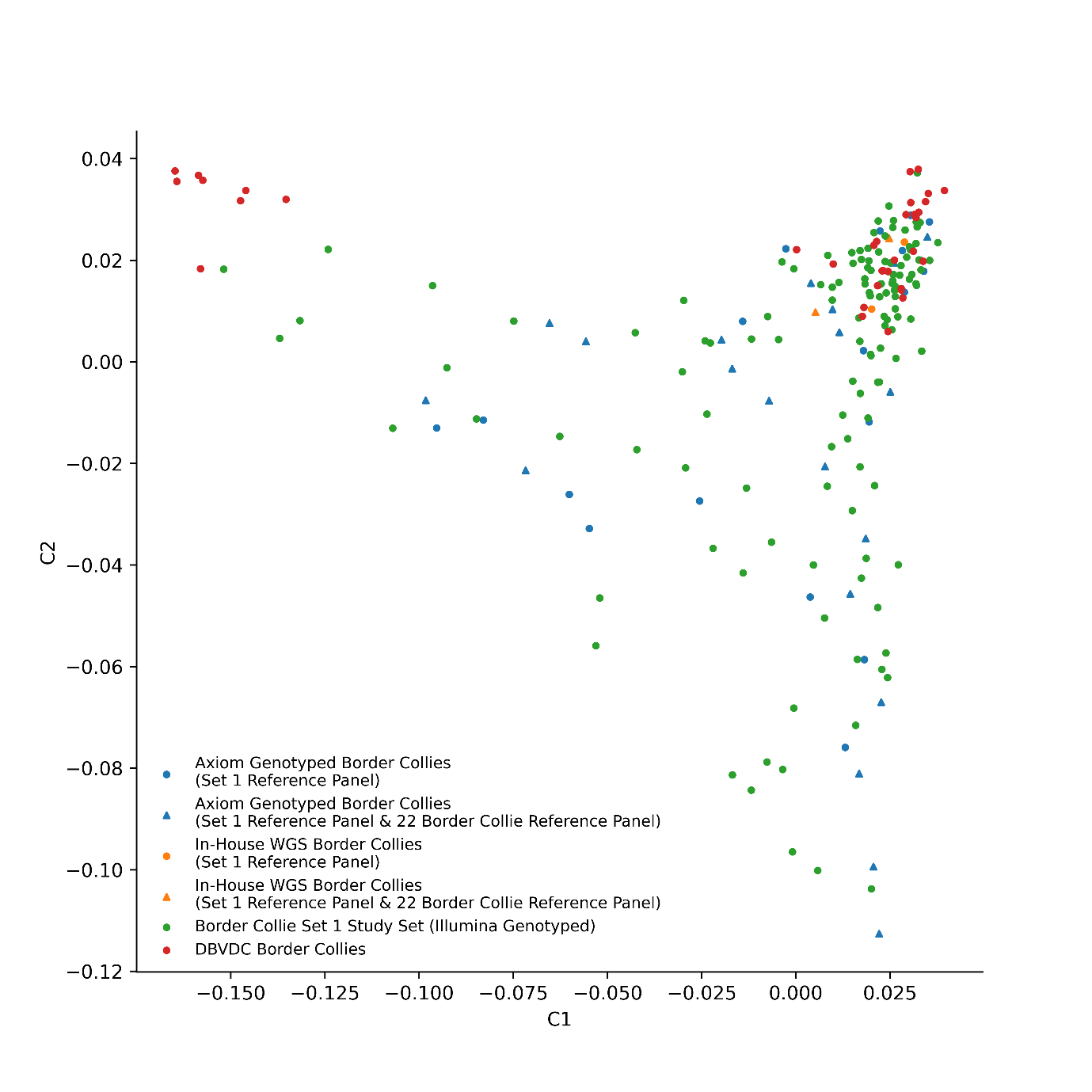


Appendix S1. Affiliations and funding information for DBVDC members

*Dog Biomedical Variant Database Consortium (DBVDC) Members:*

Gustavo Aguirre

Department of Clinical Sciences & Advanced Medicine, School of Veterinary Medicine, University of Pennsylvania, Philadelphia, United States of America

Catherine André

University of Rennes, CNRS, IGDR – UMR 6290, France

Danika Bannasch

School of Veterinary Medicine, University of California, Davis, United States of America

Doreen Becker

Institute of Genome Biology, Leibniz Institute for Farm Animal Biology (FBN), Dummerstorf, Germany

Brian Davis

College of Veterinary Medicine and Biomedical Sciences, Texas A&M University, United States of America

Cord Drögemüller

Institute of Genetics, Vetsuisse Faculty, University of Bern, Switzerland

Kari Ekenstedt

Department of Basic Medical Sciences, College of Veterinary Medicine, Purdue University, West Lafayette, Indiana, United States of America

Kiterie Faller

Royal (Dick) School of Veterinary Studies, University of Edinburgh, United Kingdom

Oliver Forman

Wisdom Health, Waltham Centre for Pet Nutrition, Leicestershire, United Kingdom

Steven Friedenberg

Department of Veterinary Clinical Sciences, College of Veterinary Medicine, University of Minnesota, St. Paul, United States of America

Eva Furrow

Department of Veterinary Clinical Sciences, College of Veterinary Medicine, University of Minnesota, St. Paul, United States of America

Urs Giger

Section of Medical Genetics (PennGen), University of Pennsylvania, Philadelphia, United States of America

Christophe Hitte

University of Rennes, CNRS, IGDR – UMR 6290, France

Marjo K. Hytönen

Department of Medical and Clinical Genetics, and Department of Veterinary Biosciences, University of Helsinki; and Folkhälsan Research Center, Helsinki, Finland

Vidhya Jagannathan

Institute of Genetics, Vetsuisse Faculty, University of Bern, Switzerland

Tosso Leeb

Institute of Genetics, Vetsuisse Faculty, University of Bern, Switzerland

Hannes Lohi

Department of Medical and Clinical Genetics, and Department of Veterinary Biosciences, University of Helsinki; and Folkhälsan Research Center, Helsinki, Finland

Cathryn S. Mellersh

Kennel Club Genetics Centre, Department of Veterinary Medicine, University of Cambridge, Cambridge, United Kingdom

James R. Mickelson

College of Veterinary Medicine, University of Minnesota, St Paul, United States of America

Leonardo Murgiano

Department of Clinical Sciences & Advanced Medicine, School of Veterinary Medicine, University of Pennsylvania, Philadelphia, United States of America

Anita Oberbauer

Department of Animal Science, University of California, Davis, United States of America

Sheila Schmutz

Department of Animal and Poultry Science, University of Saskatchewan, Saskatoon, Canada

Jeffrey J. Schoenebeck

Royal (Dick) School of Veterinary Studies, University of Edinburgh, United Kingdom

Kim M. Summers

Mater Research Institute-University of Queensland, Brisbane, Australia

Frank G. van Steenbeek

Faculty of Veterinary Medicine, Department of Clinical Sciences of Companion Animals, Utrecht University, the Netherlands

Claire Wade

School of Life and Environmental Sciences, University of Sydney, Australia

*Funding information*

Catherine André and Christophe Hitte were supported by the French Cani-DNA CRB (http://dog-genetics.genouest.org), which is part of the CRB-Anim infrastructure, ANR-11-INBS-0003. Kari J. Ekenstedt was supported by the Office of the Director, National Institutes of Health (NIH) under award number K01-OD027051. Eva Furrow was supported by the Office of the Director, National Institutes of Health (NIH) under award number K01-OD019912. Tosso Leeb was supported by the Albert-Heim Foundation (project no. 105). Kim M. Summers receives core support from the Mater Foundation, Brisbane, Australia.
